# Accurate Differentiation of *Streptococcus pneumoniae* from other Species within the *Streptococcus mitis* Group by Peak Analysis Using MALDI-TOF MS

**DOI:** 10.3389/fmicb.2017.00698

**Published:** 2017-04-25

**Authors:** Mercedes Marín, Emilia Cercenado, Carlos Sánchez-Carrillo, Adrián Ruiz, Álvaro Gómez González, Belén Rodríguez-Sánchez, Emilio Bouza

**Affiliations:** ^1^Clinical Microbiology and Infectious Diseases Department, Hospital General Universitario Gregorio MarañónMadrid, Spain; ^2^Instituto de Investigación Sanitaria Gregorio MarañónMadrid, Spain; ^3^CIBER de Enfermedades Respiratorias (CIBERES CB06/06/0058)Madrid, Spain; ^4^Medicine Department, Faculty of Medicine, Universidad Complutense de MadridMadrid, Spain; ^5^Francisco Soria-Melguizo SAMadrid, Spain

**Keywords:** *Streptococcus pneumoniae*, *Streptococcus mitis* group, routine identification, MALDI-TOF MS, peak analysis

## Abstract

Despite the benefits of MALDI-TOF MS technology (Matrix-Assisted Laser Desorption-Ionization Time-Of-Flight Mass Spectrometry) reported worldwide and the continuous improving of the available databases, discrimination between *Streptococcus pneumoniae* and closely related species within the *Streptococcus mitis* group (SMG) using this methodology has been suboptimal. However, the accurate identification at the species level of this group of microorganisms is important for the appropriate management of infected patients. In this study, 216 SMG isolates -101 *S. pneumoniae* and 115 corresponding to 7 non-pneumococcal species within this group- were analyzed. All the isolates had been previously identified by conventional methods (optochin and bile solubility tests) and non-pneumococcal isolates were confirmed by sequence analysis (*sodA* and *plys* genes) when required. The isolates were also identified with the MALDI Biotyper 3.1 (Bruker Daltonics, Bremen, Germany) using an updated library containing 6,903 Main Spectra Profiles (MSPs). All the analyzed *S. pneumoniae* were correctly identified with MALDI-TOF MS at species level using the most updated database and all the non-pneumococcal SMG isolates were also identified at the group level. Several peaks (4,964.32, 6,888.90, and 9,516.46 m/z) have been found to be specific of *S. pneumoniae*, whilst a different set of peaks have proved to be present only in *S. mitis* (6,839.07 m/z) and *S. oralis* (5,297.61, 5822.53, and 6,839.07 m/z). Peak analysis allowed correct species assignment of 101/101 S. pneumoniae isolates (100%) and 102/105 *S. mitis/oralis* isolates (97.1%). Thus, the implementation of MALDI-TOF MS plus peak analysis for the identification of this group of microorganisms may provide precise species-level information that will allow the early implementation of directed antibiotic therapy.

## Introduction

The wide implementation of MALDI-TOF MS (Matrix Assisted Laser Desorption Ionization Time-of-Flight Mass Spectrometry) has led to its use for species assignment within the viridans group streptococci (VGS) and its differentiation from *Streptococcus pneumoniae*. However, that task currently represents a challenge for the mass spectrometry techniques as well as for the molecular methods and new technologies (Doern and Burnham, 2010). Efforts to get an accurate identification of VGS species are directed to determine the clinical significance of the organism and to promptly initiate directed antibiotic treatment.

Conventional tests such as optochin sensitivity and bile solubility are still applied in laboratory routine and represent the reference method for the identification of *S. pneumoniae* isolates (Yahiaoui et al., 2016). However, *S. pneumoniae* isolates resistant to optochin have been reported in different geographical regions (Nagata et al., 2012) making this tests less than optimal also due to its low specificity (Yahiaoui et al., 2016).

The development and implementation of genotypic methods allowed a more complete view of the complexity within alpha-haemolytic Streptococci and defined six main groups of VGS: *S*. *anginosus, S. bovis, S. mitis, S. mutans, S. salivarius*, and *S. sanguinis* groups (Facklam, 2002; Doern and Burnham, 2010). DNA analysis demonstrated that several species within this group share >99% sequence homology. This is the case of *Streptococcus mitis, S. oralis* and *S. pneumoniae* (Richter et al., 2008). Thus, sequencing of housekeeping genes such as *16S rRNA* does not allow enough discrimination of these species (Hoshino et al., 2005) and different targets are required. Genes such as *tuf*, *sodA, rpoB*, or *gyrB* (Simmon et al., 2008; Póntigo et al., 2015) have been analyzed in order to determine species-specific sequences. Efforts have also been directed to the amino acid sequencing of the protein encoded by the *gyrB* gene in order to reliably identify VGS species (Galloway-Peña et al., 2014).

The availability of MALDI-TOF MS (Matrix-Assisted Laser Desorption Ionization Time of Flight Mass Spectrometry) has represented a new approach for the reliable identification of VGS at the species level. Several authors have evaluated the available MALDI-TOF instruments (Kärpänoja et al., 2014; Angeletti et al., 2015), improved samples preparation methods (Schulthess et al., 2013) and the use of peak analysis (Werno et al., 2012; Ikryannikova et al., 2013) in order to achieve accurate identification of VGS. In this study we applied an updated Biotyper database (Bruker Daltonics, Bremen, Germany) to the identification of SMG species -the most challenging species for MALDI-TOF MS to identify- from colonies grown on agar plates, without protein extraction procedures. Besides, peak analysis was performed on well characterized isolates by DNA sequencing (*sodA* and *plys* gens) in order to find putative species-specific peaks that may facilitate the identification of SMG isolates at the species level.

## Materials and methods

### Isolates and culture conditions

We analyzed a total of 216 clinical isolates of SMG from the collection of isolates identified in our microbiology laboratory between 2014 and 2016. The isolates consisted of 101 *S. pneumoniae* from blood (68), bronchial aspirates (14), conjunctival and otic exudates (5), normally sterile fluids (5), bronchoalveolar lavage (3), sputum (3), and others (3) and 115 non-pneumococcal SMG isolates from blood (52), normally sterile fluids (16), abscesses (14), urine (13), different types of biopsies (11), surgical wounds (5), and bronchial or tracheal aspirates (4). Besides, 8 reference strains were also analyzed: *S*. *anginosus* (ATCC 12395), *S. equinus* (ATCC 33317), *S. gordonii* (ATCC 33399), *S. intermedius* (ATCC 27335), *S. mutans* (ATCC 25175), *S. oralis* (ATCC 35037), *S. pneumoniae* (ATCC 27336), and *S. salivarius* (ATCC 7073). All the isolates were subcultured on Columbia agar + 5% sheep blood (Biomérieux, Marcy L'Étoile, France) and incubated at 37°C in 5% CO_2_ for 48 h.

### Conventional and genotypic identification

All the strains were identified as VGS by Gram staining, catalase test and alpha-haemolysis. Besides, the optochin susceptibility and bile solubility tests confirmed the identification of *S. pneumoniae*. On the other hand, the non-pneumococcal isolates were all confirmed by their resistance to optochin (Kellogg et al., 2001; Facklam, 2003).

DNA sequence analysis of the superoxide dismutase (*sodA*) and the pneumolysin (*plys*) genes was performed in order to provide species-level identification to non-pneumococcal isolates. A 480-bp fragment within the *sodA* gene was amplified using the degenerated primers d1 (5′'-CCITAYICITAYGAYGCIYTIGARCC-3′') and d2 (5′'-ARRTARTAIGCRTGYTCCCAIACRTC-3; Poyart et al., 1998). For the amplification of the *plys* gene, the forward primer (5′'-TGCAGAGCGTCCTTTGGTCTAT-3′') and reverse primers (5′'-TGCAGAGCGTCCTTTGGTCTAT-3′') were used in combination with a labeled probe (VIC–5′'-TGGCGCCCATAAGCAACACTCGAA-3′') as described by Corless et al. (2001). The amplified fragments were purified using the GFX PCR DNA and Gel Band Purification kit (GE Healthcare, UK) and sequenced with an AbiPrism 3,130 × l Genetic Analyzer equipment (Applied Biosystems, CA, USA). Sequences were compared with the GeneBank library using BLAST alignment software (http://www.ncbi.nlm.nih.gov/blast) and BIBI (bioInformatic bacterial identification tool: https://umr5558-bibiserv.univ-lyon1.fr/lebibi/lebibi.cgi). And the identification of the strains was carried out following the criteria established by the CLSI guidelines –(CLSI, 2008)-, with species level identification only when the homology was >99%.

Conventional methods and DNA sequencing analysis were considered as the reference methods for SMG identification.

### MALDI-TOF identification

All SMG isolates were analyzed by MALDI-TOF MS, using a Microflex LT bench top mass spectrometer (Bruker Daltonics, Bremen, Germany) as described previously –(Rodríguez-Sánchez et al., 2016)- Briefly, FlexControl 3.3 and MALDI Biotyper 3.1 software (Bruker Daltonics, Germany) was used for the acquisition of the spectra and for spectra analysis and subsequent comparison with the database (updated with 6,903 MSP entries), respectively. The available database contains 31 *S. pneumoniae*, 39 *S. mitis* and 38 *S. oralis* references. A commercial standard Bacterial Test Standard (Bruker Daltonics, Bremen, Germany) was used for calibration of the equipment and acquisition of mass spectra was performed using default settings.

Sample preparation prior to MALDI-TOF identification has been described before –(Rodríguez-Sánchez et al., 2014)-. A small amount of bacteria was transferred to a polished steel MALDI target plate using a wooden toothpick. The spots were covered with 1 μl of 100% formic acid and allowed to dry at room temperature prior to the addition of 1 μl of matrix (α-cyano-4-hydroxy-cinnamic acid solution in 50% acetonitrile and 2.5% trifluoroacetic acid) -prepared following the manufacturer's instructions-. The spots were allowed to air dry before spectra acquisition and comparison with the database. All the isolates were analyzed by MALDI-TOF MS in duplicates and the higher score value was recorded as well as the identification provided by MALDI-TOF MS. Score values for species level (≥2.0) and genus-level identification (≥1.7) used in this study are those proposed by the manufacturer. Score values below 1.7 were, accordingly, considered as not reliable. Identification agreement at the species level was considered when MALDI-TOF provided the same result as the *sodA* gene sequencing and the conventional methods. However, the identification was considered a major error when MALDI-TOF MS identified an isolate as *S. pneumoniae* whilst the conventional methods and *sodA* gene sequencing indicated that the isolate was a non-pneumococcal isolate and viceversa. When the discrepancy was between two non-pneumococcal SMG species it was considered as a minor error.

### Peak analysis

For peak analysis, 20 *S. pneumoniae*, 20 *S. mitis* and 20 *S. oralis* isolates were submitted to a standard protein extraction procedure: a 1 μl-loopful of bacteria was resuspended in 300 μl of distilled water and 900 μl ethanol, vortexed and centrifuged at maximal speed for 1 min; the pellet was then resuspended in 20 μl of 70% formic acid and the same amount of acetonitrile, vortexed again and centrifuged again for another minute at maximal speed. 1 μl of supernatant was pipetted onto the MALDI plate, allowed to dry and covered with 1 μl of matrix. The supernatant from every strain was spotted onto 6 positions. In a different spot, 1 μl of BTS was spotted on top of the dried supernatant, allowed to dry and covered with 1 μl of matrix. This position was used for calibration purposes.

FlexAnalysis 3.3 (Bruker Daltonics, Bremen, Germany) was applied for the comparison of mass spectra from the different SMG species. Spectra were smoothed, the baseline was subtracted and BTS was used as references for calibration purposes. Peaks from individual spectra were compared for strain-specific variations and spectra from all 20 isolates from the same species were considered together as a single MSP entry in order to find species-specific peaks.

The 60 isolates submitted to protein extraction were used for the characterization of species-specific peaks present in each *Streptococcus* species. Afterwards, the presence of species-specific peaks was analyzed in all *S. pneumoniae*-101-, *S. mitis*-50-, and *S. oralis*-55- isolates included in this study.

### Ethics statement

The hospital Ethics Committee approved this study and gave consent for its development. Since this study has been performed on microbiological samples, not human products, all the conditions to waive the informed consent have been met.

## Results

### Routine identification of SMG isolates with Maldi-TOF MS

During the study period, 216 SMG isolates were identified with MALDI-TOF MS as part of the clinical microbiology laboratory routine. MALDI Biotyper with 6,903 MSPs correctly discriminated *S. pneumoniae* from non-pneumococcal SMG isolates. Therefore, no major errors were detected when this database was implemented. Concordantly, all non-pneumococcal isolates were correctly identified at group level, although at the species level 14 *S. mitis/oralis* isolates were misidentified according to *sodA* sequencing analysis results (Table 1).

**Table 1:** **Isolates included in this study and their identification using MALDI-TOF MS with the Biotyper database 6903 MSPs**.

**MALDI-TOF MS/sequence analysis id**	**# Isolates**	**Biotyper 6903 MSPs**			
		**Score ≥ 2.0**	**Score ≥ 1.8**	**Score ≥ 1.6**	**MisID**
*S. gordonii*	2	2	−	−	−
*S. infantis*	1	1	−	−	−
*S. massiliensis*	1	1	−	−	-
*S. mitis*	50	43	1	1	5
*S. oralis*	55	40	6	−	9
*S. parasanguinis*	2	2	−	−	−
*S. pneumoniae*	101	83	15	3	−
*S. sanguinis*	4	4	−	−	−
Total	216	176 (81.5)	22 (10.2)	4 (1.8)	14 (6.5)

*The percentage of identifications within every score range is shown in brackets*.

Interestingly, one of the isolates selected for this study and wrongly listed as a non-pneumococcal SMG isolate was consistently identified by MALDI-TOF MS as *S. pneumoniae* (score value = 2.524). When its identification by the optochin test, the bile solubility test and *sodA* gene sequencing was double-checked it turned out that the isolate was actually a *S. pneumoniae* one, thus, confirming MALDI-TOF MS identification.

### Peak analysis

**Figure 1:**
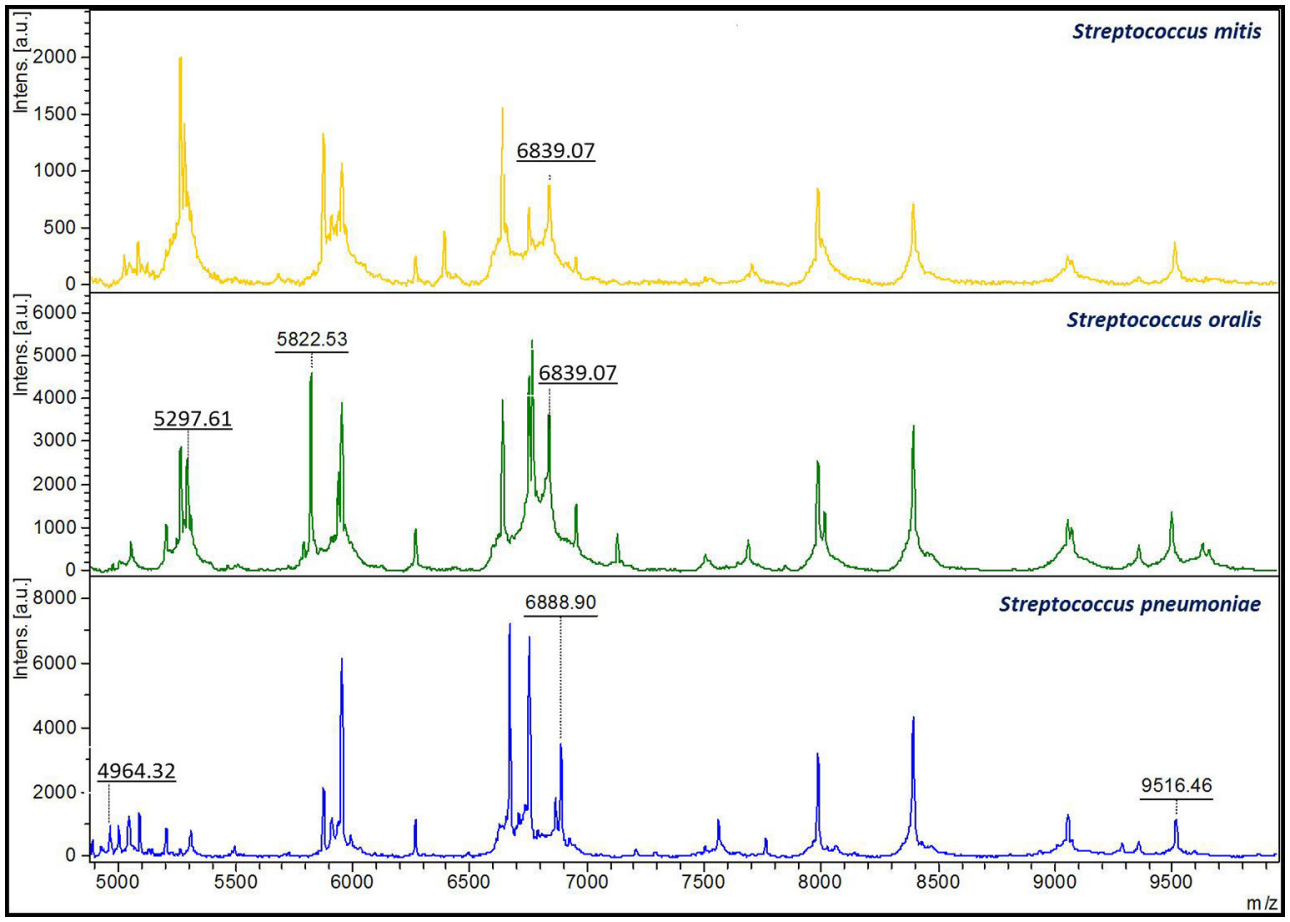
**Distinctive species-specific peaks present in close related ***Streptococcus*** species**. The 6,839.07 m/z has been shown to be present in protein spectra from *S. mitis* isolates (above). This peak is also present in *S. oralis* spectra (middle) in combination with the 5,297.61 and the 5,822.53 m/z peaks. On the other hand, *S. pneumoniae* isolates (bottom) showed three unique peaks of 4,964.32, 6,888.90, and 9,516.46 m/z.

Despite MALDI-TOF MS high level of accuracy in the identification of SMG isolates, in this study we have observed that challenging strains may still be found, leading to misidentifications. Thus, the protein spectra from the 101 analyzed *S. pneumoniae* isolates were compared with the spectra from those belonging to the *S. mitis* group, in order to single out *S. pneumoniae* specific peaks. One peak was found in at least 95.0% of the *S. pneumoniae* isolates -4,964.32 m/z- and other two peaks in 90.5% of the isolates: 6,888.90 and 9,516.46 m/z –Table 2. Their presence was also confirmed in the reference strain ATCC 27336 (Figure 1).

**Table 2:** **List of the specific peaks for ***S. pneumoniae, S. mitis***, and ***S. oralis*****.

**MALDI-TOF MS identification**	**m/z value**					
	**4964.32**	**5297.61**	**5822.53**	**6839.07**	**6888.90**	**9516.46**
***S. pneumoniae***	95.0	−	−		90.5	90.5
***S. mitis***	−	−	−	95.2	−	−
***S. oralis***	−	92.0	92.0	95.2	−	−
**Other non-pneumococcal VGS**	−	−	−	−	−	−

*None of these peaks have been detected in other VGS species. Values represent the percentage of analyzed isolates that showed these specific peaks*.

On the other hand, 92.0% of the *S. oralis* strains showed two specific peaks of 5,297.61 and 5,822.53 m/z also present in the reference strain ATCC 35037. These peaks were not detected in *S. pneumoniae* isolates neither in the other species of non-pneumococcal VGS analyzed. Besides, *S. mitis* and *S. oralis* isolates shared a peak of 6,839.07 m/z. This peak was the only species-specific feature of *S. mitis* isolates, whilst *S. oralis* displayed it together with the 5,297.61 and 5,822.53 m/z peaks (Figure 1).

Finally, although MALDI-TOF MS accurately identifies the remaining VGS groups (*S. anginosus, S. bovis, S. mitis, S. mutans, S. salivarius*, and *S. sanguinis*) and no misidentifications with *S. pneumoniae* have been reported as far as the authors know, peak comparison was performed between *S. pneumoniae* isolates and the rest of non-pneumococcal VGS, including the reference strains (data not shown). None of the *S. pneumoniae* specific peaks were found alone or in combination in the remaining non-pneumococcal VGS included in this study.

In our study, out of the 14 non-pneumococcal SMG isolates misidentified by the Biotyper system with 6,903 MSPs, all but two isolates could be correctly identified by peak analysis. The discrepant identifications corresponded to two *S. mitis* isolates identified by *sodA* sequencing analysis that were identified as *S. oralis* by the updated database and by peak analysis –Table 3. Further genomic targets should be analyzed in order to resolve these discrepancies.

**Table 3:** **Reidentification by peak-analysis of SMG isolates that showed discrepancies in the species assignment by MALDI-TOF and the conventional or the molecular methods**.

**Isolate number**	**Conventional methods/sequence analysis Id**	**Biotyper 6,903 MSPs**	**Score**	**Peak analysis (m/z)**						**ID by peak analysis**
				**4964.32**	**5297.61**	**5822.53**	**6839.07**	**6888.90**	**9516.46**	
19	*S. oralis*	*S. mitis*	2.38	−	+	+	+	−	−	*S. oralis*
26	*S. oralis*	*S. mitis*	2.26	−	−	+	+	−	−	*S. oralis*
35	*S. oralis*	*S. mitis*	2.50	−	+	+	+	−	−	*S. oralis*
41	*S. mitis*	*S. oralis*	2.18	−	−	−	+	−	−	*S. mitis*
42	*S. mitis*	*S. oralis*	2.33	−	−	−	+	−	−	*S. mitis*
46	*S. mitis*	*S. oralis*	2.24	−	−	−	+	−	−	*S. mitis*
47	*S. mitis*	*S. oralis*	2.22	−	−	+	+	−	−	*S. oralis*
51	*S. mitis*	*S. oralis*	2.33	−	+	+	+	−	−	*S. oralis*
54	*S. oralis*	*S. mitis*	2.24	−	+	+	+	−	−	*S. oralis*
57	*S. oralis*	*S. mitis*	1.84	−	+	+	+	−	−	*S. oralis*
58	*S. oralis*	*S. mitis*	2.28	−	+	+	+	−	−	*S. oralis*
59	*S. oralis*	*S. mitis*	2.36	−	−	+	+	−	−	*S. oralis*
60	*S. oralis*	*S. mitis*	2.36	−	−	+	−	−	−	*S. oralis*
64	*S. oralis*	*S. mitis*	2.32	−	+	+	+	−	−	*S. oralis*

*In all but two cases the identification provided by the reference methods was confirmed by peak analysis*.

In summary, the updated database allowed the correct species-level identification of 100% of the *S. pneumoniae* isolates and 100% of the non-pneumococcal isolates at the group-level. Besides, the performance of peak analysis confirmed the identification of all *S. pneumoniae* isolates and provided a correct species-level discrimination of 103/105 *S. mitis* and *S. oralis* isolates (98.1%).

## Discussion

The identification of SMG isolates with MALDI-TOF MS has been improved in the last years thanks to the availability of updated databases. In this study, 100% of the *S. pneumoniae* and 87.8% of the non-pneumococcal SGM isolates were correctly identified at the species level using an updated database. Similar studies have reported 100% accuracy of the Biotyper system with 5,267 entries in the identification of *S. pneumoniae* isolates, but only 61.0–66.7% species-level correct identification of non-pneumococcal VGS isolates and up to 23.4% of misidentification of these species as *S. pneumoniae* (Angeletti et al., 2015; Chen et al., 2015). Another study that applied the Biotyper database updated with 5,989 entries for the identification of VGS species reported 21 isolates of S. *mitis/oralis/pseudopneumoniae* out of 107 misidentified (19.6%) –(Zhou et al., 2016)-. These results are clearly far from the 14 misidentified isolates (6.5%) observed in our study.

A recent paper by Harju et al. (2017) has reported 100% correct species assignment to *S. pneumoniae* isolates and 99.0% correct group level identification of non-pneumoccocal isolates using the Biotyper library with 5,627 MSPs. By applying an algorithm that assigns a numerical value to each of the 10 identifications in the ranking list provided by the Biotyper software, the authors achieved 100% correct group-level identification of non-pneumococcal isolates.

In our study, 100% of the *S. pneumoniae* isolates were correctly identified at the species level using a more updated library and their identification was confirmed by peak analysis. Besides, group-level identification of non-pneumococcal isolates was obtained in 100% of the cases. Peak analysis of these isolates allowed species-level identification of 103/105 *S. mitis* and *S. oralis* isolates (98.1%) according to DNA sequencing analysis. Although these results may have little clinical value, the detection of the species-specific peaks reported in this study may provide important epidemiological information.

Peak analysis has been applied before for confirmation of MALDI-TOF MS identification (Werno et al., 2012; Ikryannikova et al., 2013; Chen et al., 2015). The peaks found by Werno et al. for the specific detection of *S. pneumoniae* (2,937.5 and 5,877 m/z) and *S. mitis/oralis* (2,911, 5,824, and 6,955 m/z) have been applied in our laboratory for routine discrimination of *S. pneumoniae* from non-pneumococcal VGS strains. However, none of the peaks propose by Ikryannikova et al. (2013) and Chen et al. (2015) for species discrimination was found in our isolates. The peak of 5,824 m/z described by Werno et al. as specific for *S. mitis/oralis* is in agreement with the 5,822.53 m/z found in our study as *S. oralis* specific. The species specific peaks described in this study allowed the confirmation of MALDI-TOF MS identification in 214/216 cases. Peak analysis enabled the correct identification of 12/14 *S. mitis/oralis* isolates misidentified by MALDI-TOF MS.

Thus, we can conclude that the improvement of the Biotyper database allows an accurate identification of SMG isolates from single colonies. However, this group of microorganisms contains highly similar species which pose a challenge for DNA- and protein-based diagnostic methods. For instance, Biotyper database with 6,903 entries contains only 5 representative spectra of *S. pseudopneumoniae*. Therefore, the chances to correctly identify this species using MALDI-TOF MS are scarce, and so were the possibilities to identify *S. pseudopneumoniae* by sequencing the *sodA* and the *plys* genes.

Despite the fact that the updated database misidentified a high number of *S. mitis/oralis* isolates (13.3%), peak analysis may allow a correct species assignment. Besides, this kind of misidentification is considered a minor error that does not hamper the early implementation of directed antibiotic therapy. Thus, our recommendation is to apply MALDI-TOF MS for the identification of *S. pneumoniae* and SMG isolates from single colonies and perform peak analysis only for *S. mitis/oralis* isolates for which both identifications alternate in the ranking list provided by MALDI-TOF MS and those that obtain low score values or to confirm the identity of *S. pneumoniae* isolates. The implementation of this approach may also save extra costs derived from the need to sequence the SGM isolates in order to get a final identification.

## Author contributions

MM carried out the sequencing of the isolates; EC performed the conventional methods for bacterial identification, discussed the results and reviewed the manuscript; CS collaborated in the design of the study and reviewed the manuscript; AR performed the analysis of the isolates by MALDI-TOF and analyzed the protein spectra; AG assisted in the spectra analysis; EB reviewed and discussed the manuscript and BR collaborated in the design of the study, the spectra analysis, the discussion of the results and the manuscript writting.

### Conflict of interest statement

The authors declare that the research was conducted in the absence of any commercial or financial relationships that could be construed as a potential conflict of interest.
